# Antioxidant Potential of Bark Extracts from Boreal Forest Conifers

**DOI:** 10.3390/antiox2030077

**Published:** 2013-07-11

**Authors:** Jean Legault, Karl Girard-Lalancette, Dominic Dufour, André Pichette

**Affiliations:** Laboratoire d’analyse et de séparation des essences végétales (LASEVE), Département des Sciences fondamentales, Université du Québec à Chicoutimi (UQAC), 555, boulevard de l’Université, Chicoutimi, Québec G7H 2B1, Canada; E-Mails: karl.lalancette@gmail.com (K.G.-L.); dominicdufour@gmail.com (D.D.); andre.pichette@uqac.ca (A.P.)

**Keywords:** phenolic content, ORAC, antioxidant cell-based assay, *Pinus banksiana*, *Pinus resinosa*, *Pinus strobus*, *Picea glauca*, *Picea mariana*, *Larix laricina*, *Abies balsamea*

## Abstract

The bark of boreal forest conifers has been traditionally used by Native Americans to treat various ailments and diseases. Some of these diseases involve reactive oxygen species (ROS) that can be prevented by the consumption of antioxidants such as phenolic compounds that can be found in medicinal plants. In this study, ultrasonic assisted extraction has been performed under various solvent conditions (water:ethanol mixtures) on the bark of seven boreal forest conifers used by Native Americans including: *Pinus strobus*, *Pinus resinosa*, *Pinus banksiana*, *Picea mariana*, *Picea glauca*, *Larix laricina*, and *Abies balsamea*. The total phenolic content, as well as ORAC_FL_ potency and cellular antioxidant activity (IC_50_), were evaluated for all bark extracts, and compared with the standardized water extract of *Pinus maritima* bark (Pycnogenol), which showed clinical efficiency to prevent ROS deleterious effects. The best overall phenolic extraction yield and antioxidant potential was obtained with *Picea glauca* and *Picea mariana*. Interestingly, total phenolic content of these bark extracts was similar to Pycnogenol but their antioxidant activity were higher. Moreover, most of the extracts did not inhibit the growth of human skin fibroblasts, WS1. A significant correlation was found between the total phenolic content and the antioxidant activity for water extracts suggesting that these compounds are involved in the activity.

## 1. Introduction

Native Americans used the plants of the boreal forest to treat various ailments and diseases [1]. The bark of several conifers, such as *Pinus strobus* (white pine), *Picea glauca* (white spruce), *Larix laricina* (Larch tamarack), and *Abies balsamea* (balsam fir) was used. The bark was prepared as decoctions, infusions or poultices to treat gonorrhea, tuberculosis, diarrhea, pain, cold, kidney troubles, burns, inflammation, and rheumatoid arthritis [1,2,3,4,5,6]. However, bark was mainly used for cough relief [1,2,7]. Moreover, some species were used as an expectorant to treat breathing difficulties (*Pinus strobus*, *Picea glauca*), and persistent cough (*Larix laricina*) [1].

Reactive oxygen species (ROS) have been implicated in several diseases and symptoms for which conifer bark was used, in particular inflammation [8,9] and rheumatoid arthritis [10]. ROS also play a role in chronic obstructive pulmonary diseases (COPD) [11,12]. The COPD are characterized by the secretion of mucus, breathing difficulties and persistent coughing. Interestingly, the consumption of polyphenolic compounds showed to be beneficial to relieve COPD [13,14,15,16]. The water extract of *Pinus maritima* bark (pycnogenol) is rich in phenolic compounds, mainly procyanidins and phenolic acids, and possesses a strong free radical-scavenging activity against ROS [17]. Pycnogenol has been reported to increase plasma antioxidant capacity and to significantly improve pulmonary functions and asthma symptoms [18,19]. In spite of the strong potential of bark extracts from boreal forest conifers, the total phenolic content and the antioxidant activity for several of them are poorly studied. In 2009, Diouf *et al.*, reported antioxidant and anti-inflammatory activities of hot water extract from *Picea mariana* bark [20]. In addition, Garcia-Pérez *et al.*, (2010) reported antioxidant and anti-proliferative properties of bark extracts from *Picea mariana* on normal and psoriatic keratinocytes [21]. More recently, Royer *et al.*, (2013) suggested that *Pinus banksiana* and *Picea mariana* barks possess an anti-aging potential due to their antioxidant, anti-enzymatic and antimicrobial activities [22].

In this study, we report the total phenolic content of bark extracts from seven *pinaceae* of the boreal forest, including *Pinus banksiana* (Lamb.), *Pinus resinosa* (Aiton), *Pinus strobus* (L.), *Picea glauca* (Moench), *Picea mariana* (Mill), *Larix laricina* (Du Roi) and *Abies balsamea* (L.). The antioxidant activity of conifer bark extracts was also evaluated using ORAC_FL_ and a cell-based antioxidant assay. The results are compared with Pycnogenol, a standardized bark extract of *Pinus maritima*.

## 2. Experimental Section

### 2.1. Chemicals

6-Hydroxy-2,5,7,8-tetramethylchroman-2-carboxylic acid (97%) (Trolox), 2,2′-Azobis(2-methylpropionamidine) dihydrochloride (97%) (AAPH), Folin-Ciocalteu phenol reagent (FC), sodium carbonate decahydrate (99.5%), Fluoresceine disodium salt (FL), dichlorofluorescin diacetate (97%) (DCFH-DA), *tert*-butyl hydroperoxyde (70%) (*t*-BuOOH), resazurin (92%) (Rz), and Hoescht (95%) (Ho), were all purchased from Sigma-Aldrich (Oakville, Ontario, Canada). Pycnogenol was produced by Swiss Herbal Remedies Ltd. (Richmond Hill, Ontario, Canada), with standardized potency of 85% proanthocyanidins. Pycnogenol tablets contain 25 mg of *Pinus maritima* extract and also contain dicalcium phosphate, microcrystalline cellulose and magnesium stearate. Tablets were grinded and extracted 1 h with 5 mL of water.

### 2.2. Plant Material and Preparation of Crude Bark Extract

All conifer bark specimens were harvested in June 2005 near station Simoncouche in the Réserve faunique des Laurentides, Québec, Canada. The specimens were identified by Patrick Nadeau, and deposited to herbarium of Saguenay-Lac-Saint-Jean (Département des Sciences Fondamentales; Université du Québec à Chicoutimi). Barks were dried at room temperature then powdered and stored at −20 °C. All extractions were ultrasonic assisted using a Sonifier Cell disruptor 350 (BRANSON Ultrasonics Corporation), with output control set at 7/10, and performed with 25 g of bark powder in 375 mL of solvent for 30 min. Extraction mixtures were constantly mixed with a magnetic agitator and were maintained at 30 °C. Each bark sample was extracted in parallel with five different solvent conditions: ethanol:water [0:100], [25:75], [50:50], [75:25], [100:0]. Extraction mixtures were then filtered and dried under vacuum at room temperature for 3 days up to constant weight.

### 2.3. Dosing of Total Phenol Content

The total phenolic content was determined using the Folin-Ciocalteu reagent according to the procedure reported by Singleton and Rossi [23], with some modifications. Briefly, a volume of 50 μL containing growing concentrations of extract ranging from 0.39 to 50 mg/mL were mixed with 25 μL of 1:2 water diluted Folin-Ciocalteu reagent in transparent flat-bottom 96-well plates (*NUNC*). All manipulations were performed in a light shielded environment. After 5 min of reaction, 125 μL of sodium carbonate decahydrate solution (20 g/100 mL) was added to each well. Absorbance was then measured at 758 nm using an automated Varioskan Ascent plate reader (*Thermo Electron*). Analysis was performed in triplicate, and the results were expressed in gallic acid equivalents.

### 2.4. ORAC_FL_ Assay

The procedure was modified from the method described by Ou *et al.* [24]. Briefly, the ORAC assay was carried out in black round-bottom 96-well plate (Costar) on a Fluoroskan Ascent Fl™ plate reader (*Labsystems*). Trolox was used as a control standard. The experiment was conducted at 37.5 °C and pH 7.4, with a blank sample in parallel. The fluorimeter was programmed to record the fluorescence of fluorescein every 60 s after addition of 2,2′-azobis (2-amidinopropane) dihydrochloride (AAPH). The final results were calculated by comparing the net areas under the fluorescein decay curves between the blank and the samples. ORAC values were expressed in micromoles of Trolox equivalents (TE) per milligram (μmol TE/mg).

### 2.5. Cell Culture

The murine fibrosarcoma L-929 (ATCC #CCL-1) and human skin fibroblasts WS1 (ATCC # CRL-1502) cell line were obtained from the American Type Culture Collection (ATCC, Manassas, USA). They were grown in Minimum Essential Medium with Earle’s salts supplemented with 10% fetal calf serum (Hyclone, Logan, USA), solution of vitamins (1×), sodium pyruvate (1×), non-essential amino acids (1×), penicillin (100 IU) and streptomycin (100 μg/mL) (Mediatech Cellgro^®^). Cells were cultured in a humidified atmosphere at 37 °C in 5% CO_2_.

### 2.6. Antioxidant Cell-Based Assay

Antioxidant activity was evaluated using the DCFH-DA assay as described previously [25], with some modifications. Briefly, L-929 cells were plated in 96-well plates at 10,000 cells per well and incubated for 24 h at 37 °C and 5% CO_2_. The cells were washed with 150 μL Hank’s balanced salt solution (HBSS) at pH 7.4 and incubated for 30 min with 100 μL HBSS (pH 7.4) containing 5 μM DCFH-DA. The cells were then washed again with 150 μL HBSS. To assess the antioxidant activity, the cells were incubated with a growing concentration of extract (0.2–200 μg/mL), in the absence or presence of 200 μM *tert*-butylhydroperoxide (*t*-BuOOH). The final concentration of extraction solvent in the culture medium was maintained at 0.5% (volume/volume) to avoid solvent toxicity. Fluorescence was measured after 1 h and 4 h on the automated plate reader (Fluoroskan Ascent FL™, Labsystems) using an excitation wavelength of 485 nm and an emission wavelength of 530 nm. Antioxidant activity is expressed as the concentration of extract inhibiting 50% of DCFH oxidation (IC_50_).

### 2.7. Cytotoxicity Assay

Exponentially growing WS1 cells were plated in flat-bottom 96-well microplates (Costar, Corning Inc.) at a density of 5 × 10^3^ cells per well in 100 μL of culture medium and were allowed to adhere for 16 h before treatment. Increasing concentrations of extract (1.5–200 μg/mL) in their respective extraction solvent were then added (100 μL per well). The final concentration of ethanol in the culture medium was maintained at 0.5% (volume/volume) to avoid solvent toxicity. The cells were incubated for 24 h in the presence or absence of bark extract. Cytotoxicity was assessed using the resazurin reduction test [26]. Fluorescence was measured on an automated Fluoroskan Ascent FL™ plate reader (Labsystems) using excitation and emission wavelengths of 530 nm and 590 nm, respectively. Cytotoxicity is expressed as the lowest concentration of tested extract inhibiting 20% or more of cell growth in comparison with untreated cells.

### 2.8. Statistical Analysis

Data were expressed as means ± standard deviation from at least three determinations (*n* ≥ 3). Comparisons between groups were performed using Kruskal-Wallis one way analysis of variance on ranks, with pairwise comparison by Student-Newman-Keuls method. P values of 0.05 or less were considered as statistically significant. Relationship between ORAC, IC_50_ and total phenolic content were determined using Pearson correlation, followed by linear regression. All statistical analysis were done with SigmaStat 3.5 and Microsoft Excel.

## 3. Results and Discussion

In this study, various extracts of bark from boreal forest conifers were evaluated for their total phenolic content and antioxidant potency. The tested conifers were: *Pinus banksiana*, *Pinus resinosa*, *Pinus strobus*, *Picea glauca*, *Picea mariana*, *Larix laricina* and *Abies balsamea*. The antioxidant activity and total phenolic content were compared with a standardized water extract of *Pinus maritima* bark (pycnogenol) recognized as having a strong antioxidant activity. The main constituents of pycnogenol are phenolic compounds, including monomers (catechin, epicatechin and taxifolin), and condensed flavanoids (procyandins and proanthocyanidins) [17]. Pycnogenol also contains phenolic acids, such as caffeic, ferulic, and p-hydroxybenzoic acids [17].

### 3.1. Extraction Yield, Total Phenol Content and Cytotoxicity of Various Conifer Bark Extracts

The bark of each conifer was extracted using sonication with five solvent conditions including water:ethanol [100:0]; [75:25]; [50:50]; [25:75]; [0:100]. The total extraction yields, presented in Table 1, show that the extracted quantity ranged from 5 to 30 g for 100 g of powdered bark. The total phenolic content of bark extracts were evaluated using Folin-Ciocalteu assay. This method allows to measure phenolic and polyphenolic compounds such as phenolic acids, flavonoids, and tannins. The results, expressed as grams of total phenolic compounds (gallic acid equivalent) for 100 g of extract, are presented in Table 1. Pycnogenol was used as a positive control with total gallic acid equivalent phenolic content of 48 ± 5 g per 100 g of extract while Ustun *et al.*, 2012 reported a concentration of about 57 g/100 g [27]. Of all the tested species of conifer barks, *Pinus resinosa-*[50:50], *Picea glauca-*[100:0] and *Picea mariana-*[75:25] were found the richest in phenolic compounds with respectively 61 ± 4 g, 59 ± 5 g and 55 ± 6 g per 100 g of extract. These values are similar to Pycnogenol. For the other tested species, the highest total phenol content measured in the extracts were in the following order: *Pinus banksiana-*[25:75] (45 ± 1 g/100 g), *Larix laricina-*[100:0] (34 ± 2 g/100 g), *Abies balsamea-*[50:50] (32 ± 2 g/100 g) and *Pinus strobus-*[75:25] (26 ± 1 g/100 g). The cytotoxicity of all bark extracts was evaluated on human skin fibroblast WS1. The cells were incubated in the presence or absence of growing concentrations of extracts for 24 h. In Table 1, the results show that all extracts, with the exception of the *Larix laricina-*[75:25] extract, do not inhibit cell growth in doses of 50 μg/mL and less, indicating that the extracts are not cytotoxic for WS1 in these dose ranges. For the *Picea mariana* and *Pinus banksiana* extracts, no cytotoxicity was found in doses as high as 100 to 200 μg/mL. Royer *et al.*, reported no cytotoxicity of hot water extract of *Picea mariana* on normal keratinocytes at concentrations lower than 55 μg/mL [21].

The extraction yields and phenolic contents of bark extracts from three species of this study (*Abies balsamea*, *Pinus banksiana*, *Picea mariana*) has been reported previously by another research group which used different extraction methods such as hot water reflux and ethanol maceration [21,22]. In comparison with their results, the overall ultrasonic assisted bark extraction yield obtained with *Abies balsamea* and *Pinus banksiana*, considering all water-ethanol extraction conditions, has been lower than yields they obtained using 1 h hot water reflux but similar to ethanol maceration [21,22]. However, ultrasonic assisted *Picea mariana* bark extraction resulted in higher extraction yield than both other methods. In terms of phenolic content richness, short ultrasonic assisted extraction provides better recovery than hot water reflux and ethanol maceration, except for *Abies balsamea* which result in lower total phenolic content [21,22].

**Table 1:** Total extraction yield, extract phenolic content and cytotoxicity.

Conifer species	Extraction conditions (water:ethanol)	Extraction yield ^a^ (g/100 g)	Extract Phenolic content (g GAE/100 g) ^b^	Cytotoxicity (μg/mL) ^c^
*Pinus banksiana*	100:0	9	27 ± 2	>100
	75:25	11	30 ± 2	>100
	50:50	12	37 ± 4	>100
	25:75	13	45 ± 1	>200
	0:100	15	23 ± 1	>100
*Pinus resinosa*	100:0	5	37 ± 4	>200
	75:25	7	61 ± 6	>100
	50:50	7	61 ± 4	>100
	25:75	11	46 ± 3	>100
	0:100	10	42 ± 5	>50
*Pinus strobus*	100:0	7	21 ± 3	>50
	75:25	9	26 ± 1	>200
	50:50	15	22 ± 1	>50
	25:75	9	15 ± 2	>50
	0:100	9	10 ± 1	>50
*Picea glauca*	100:0	17	59 ± 5	>100
	75:25	21	51 ± 4	>100
	50:50	24	55 ± 9	>50
	25:75	29	36 ± 4	>200
	0:100	26	48 ± 5	>100
*Picea mariana*	100:0	14	50 ± 5	>200
	75:25	17	55 ± 6	>100
	50:50	23	48 ± 4	>200
	25:75	24	46 ± 5	>200
	0:100	23	43 ± 3	>100
*Larix laricina*	100:0	11	34 ± 2	>50
	75:25	15	27 ± 2	>12.5
	50:50	24	29 ± 3	>50
	25:75	30	26 ± 3	>50
	0:100	30	29 ± 3	>50
*Abies balsamea*	100:0	7	6 ± 1	>100
	75:25	9	21 ± 1	>100
	50:50	7	32 ± 2	>100
	25:75	17	21 ± 2	>50
	0:100	20	20 ± 1	>50

^a^ Total extraction yield from 100 g of dry powdered conifer bark. ^b^ Grams of total phenolic compounds (gallic acid equivalent) for 100 g of extract. ^c^ The concentration of conifer bark extract (μg/mL) is considered cytotoxic when it inhibits cell growth >20% in comparison with untreated cells.

### 3.2. Evaluation of Antioxidant Activity of Extracts Using ORAC_FL_ and Cell-Based Assay

**Figure 1:**
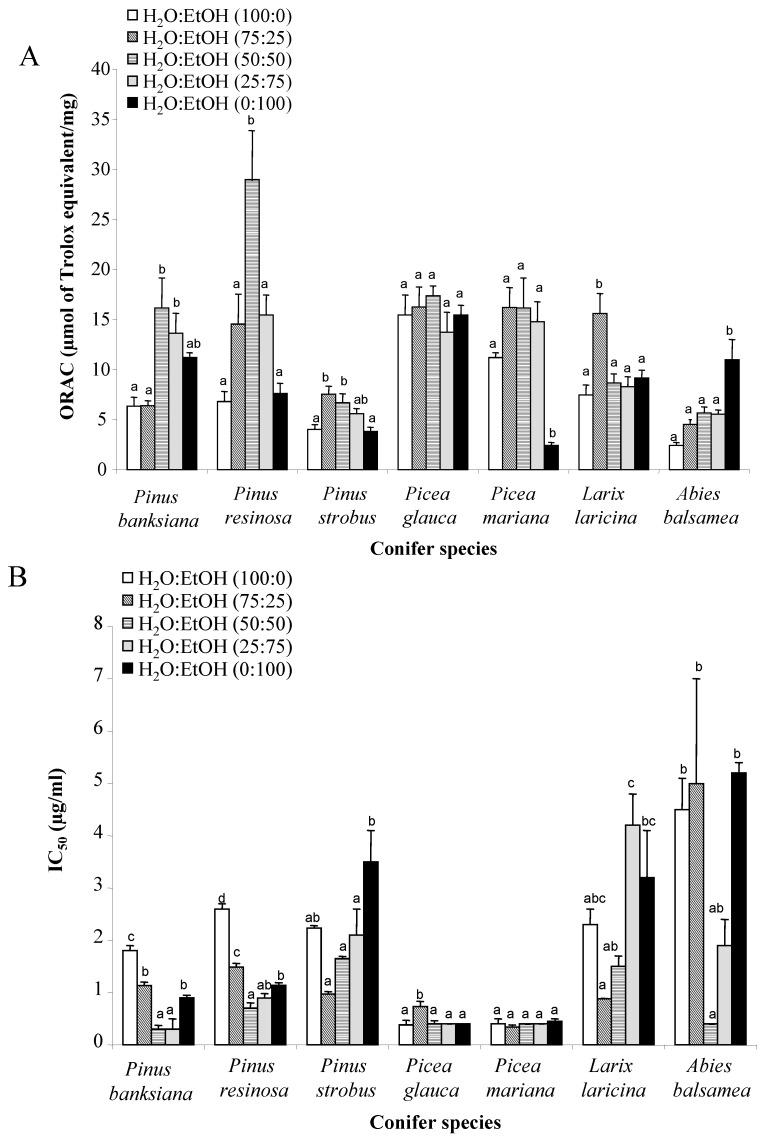
Antioxidant activity of conifer bark extracts using (**A**) ORAC assay and (**B**) cell-based assay. ORAC values are expressed as micromoles of Trolox equivalent per milligram of dry extract. IC_50_ values obtained using cell-based assay were expressed as the concentration inhibiting fifty percent of DCFH oxidation induced by *tert*-butyl hydroperoxide. The ORAC value and IC_50_ of the positive control, pycnogenol, are 5.4 ± 0.3 μmol TE/mg and 1.5 ± 0.2 μg/mL respectively. All assays were conducted in triplicate, and the mean values are used. The vertical bars represent the standard deviation of each data point. Means within each group with different letters (a–c) differ significantly (*p* < 0.05) from each other.

The antioxidant activity of bark extracts was evaluated using ORAC and a cell-based assay. ORAC_FL_ values are expressed as Trolox equivalent (μmol) by mg of extracts, and cell-based assays are expressed as the concentration inhibiting fifty percent (IC_50_) of *tert*-butylhydroperoxide (*t*-BuOOH) induced oxidation of 2′,7′-dichlorofluorescin (DCFH). Pycnogenol was used as a positive control with an ORAC_FL_ value of 5.4 ± 0.3 μmol TE/mg and an IC_50_ of 1.5 ± 0.2 μg/mL. This result is slightly lower than ORAC values (6.5 to 7.7 μmol TE/mg) reported by Dudonné *et al.*, 2009 [28]. Results presented in Figure 1 show that ORAC_FL_ values of all extracts ranged from 2.4 to 29 μmol TE/mg, while cell-based assay IC_50_ values ranged from 5.2 to 0.3 μg/mL. The *Pinus resinosa-*[50:50] possesses the highest ORAC_FL_ value with 29 μmol TE/mg. According to the cell-based assay, the results show that the extracts of *Pinus banksiana-*[50:50] and [25:75], *Picea mariana-*[75:25] and *Picea glauca-*[100:0], have a higher antioxidant activity with an IC_50_ smaller than 0.4 μg/mL. The correlation between ORAC_FL_ and cell-based assay for all species is weak but significant (*P* < 0.05) with a coefficient of determination *R*^2^ of 0.24. This weak correlation between both methods can possibly be explained by the cellular context of cell-based assay which is not present in ORAC assay.

The antioxidant activity of bark extracts was analysed for each species in order to evaluate the best conditions of extraction. For *Pinus banksiana*, the water:ethanol extracts [50:50] and [25:75] possess the highest antioxidant activity in comparison with other conditions of extraction. The ORAC_FL_ values for *Pinus banksiana-*[50:50] and [25:75] are of 16 ± 3 μmol TE/mg and 14 ± 2 μmol TE/mg, respectively, and their IC_50_ are of 0.30 ± 0.07 and 0.3 ± 0.2 μg/mL. Both extracts contain higher gallic acid equivalent phenolic concentrations with 37 g/100 g for [50:50] extract and 45 g/100 g for [25:75] extract. The most active extract of *Pinus resinosa* is the water:ethanol [50:50] with an ORAC value of 29 ± 5 μmol TE/mg and an IC_50_ of 0.7 ± 0.1. This extract is very rich in phenolic compounds, with 61 ± 4 g GAE/100 g, suggesting that phenolic compounds are in part responsible for the antioxidant activity. However, the [75:25] extract is also rich in phenolic compounds (61 ± 5 g/100 g) but significantly less active than the [50:50] extract. For *Pinus strobus*, no significant difference was found between the three most active conditions which include the water:ethanol [75:25], [50:50], and [25:75], with ORAC values ranging from 5.0 to 8.5 μmol TE/mg and IC_50_ ranging from 0.91 to 2.7 μg/mL. The total phenolic content is significantly correlated with cell-based assay but not with ORAC_FL_ values. The extracts of *Picea glauca* and *Picea mariana* possess similar antioxidant activities, using both assays. Indeed, the ORAC_FL_ values ranged from 11 to 17 μmol TE/mg and the IC_50_ ranged from 0.3 to 0.73 μg/mL. The total phenolic contents of these extracts were also relatively high with concentrations ranging from 36 g to 59 g GAE/100 g. The *Larix laricina*-[75:25] possesses a significantly higher antioxidant activity in comparison with other conditions of extraction. For this extract, the ORAC_FL_ value is 16 μmol TE/mg and the IC_50_ is of 0.878 μg/mL. However, the total phenolic content for all conditions tested are not significantly different with values ranging from 27 ± 2 g GAE to 34 ± 2 g GAE/100 g. For *Abies balsamea*, the highest antioxidant activity was obtained with water:ethanol [50:50] according to cell-based assay with IC_50_ of 0.3 μg/mL. This extract is also significantly richer in phenolic compounds (32 g GAE/100 g) in comparison with other conditions of extraction. In contrast, the highest ORAC_FL_ value was obtained with a [0:100] extract (11 μmol TE/mg) which contained 20 g GAE of phenolic compounds by 100 g of extract.

Altogether, these results indicate that the total phenolic content do not always correlate with antioxidant activity. Therefore, the relationship between total phenolic content and ORAC_FL_ and cell-based assay were analyzed.

### 3.3. Relationship between Total Phenol Content and Antioxidant Activities

**Figure 2:**
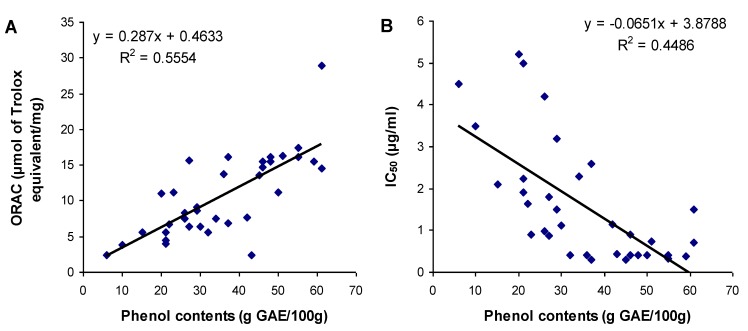
Relationship between (**A**) ORAC values and (**B**) IC_50_ values of extracts of conifer bark, and their content in phenolic compounds. Solid lines represent linear regression curves. The regression coefficient (*R*^2^) and the equation of curves are given.

The relationship between the total gallic equivalent phenolic contents and the antioxidant activity (ORAC_FL_ and cell-based assay) was determined by a Pearson correlation analysis for all conifer barks and conditions of extraction. Significant but moderate correlations were found between phenolic contents and ORAC as well as phenolic contents and cell based IC_50_ results, with correlation coefficient of *r* = 0.745 (*P* < 0.001) and *r* = −0.670 (*P* < 0.001) respectively. Once the correlation was established between phenolic contents and antioxidant activities, their relation was described using linear regression analyses (Figure 2). The results show that even though phenolic content and antioxidant activity are related, their modest correlation and regression coefficients indicate that phenolic content may not explain totally the antioxidant activity when considering all extraction conditions. This suggests a change in the chemical composition of the extracts according to the solvent mixture used. Garcia-Perez *et al.* reported that the extracts obtained using ethanol maceration in comparison with hot water reflux contain lower total phenol content and higher concentration of hydroxycinnamic acids and proanthocyanidins [21]. In addition, antioxidant activity of ethanol extract was found decreased in comparison with hot water extract.

**Figure 3:**
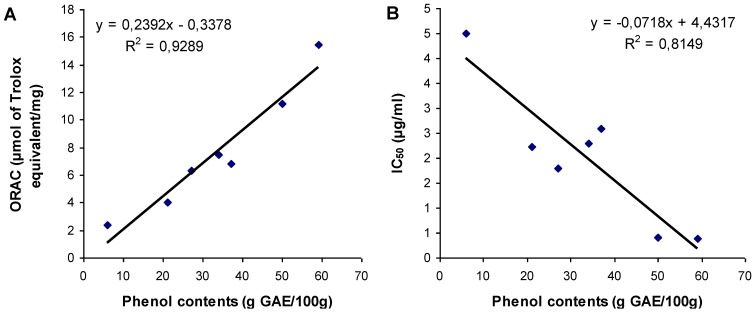
Relationship between (**A**) ORAC values and (**B**) IC_50_ values of all water extracts of conifer bark, and their content in phenolic compounds. Solid lines represent linear regression curves. The regression coefficient (*R*^2^) and the equation of curves are given.

Native Americans used water infusion of conifer bark to relieve various ailments and diseases [1]. Therefore, water soluble phenolic compounds could be responsible for the activity of conifer bark. The relationship between total phenolic contents and activity of the bark extracts was determined again with Pearson correlation for all conifer species but this time including only water extracts. Both ORAC activity and cellular IC_50_ results were strongly correlated with phenolic contents, with a correlation coefficient of *r* = 0.9640 (*P* < 0.001) and *r* = −0.9030 (*P* = 0.005) respectively. Both relations are shown by linear regression in Figure 3. These good correlations suggest that phenolic compounds could explain the activity of water extracts of all conifer species better than the activity measured including all extraction conditions. Also, the slightly lower correlation coefficient obtained with cellular assay results, in comparison with the correlation coefficients obtained with ORAC, corroborate the presumption that cellular context may measure activities that are not measurable with the strictly chemical ORAC assay.

Considering only water extracts, *Picea glauca* and *Picea mariana* extracts possess the most significant (*P* < 0.05) total phenolic content and antioxidant activities in comparison with other species. Garcia-Pérez *et al.*, also reported that hot water extract of *Picea mariana* bark are richer in phenolic compounds and also more antioxidant than bark extracts of *Abies balsamea* and *Pinus banksiana* [21]. In addition, Garcia-Pérez *et al.*, have isolated and characterized most bioactive polyphenols in hot water extract of *Picea mariana* bark including trans-resveratrol which has a strong antioxidant activity [29]. On the other hand, water extracts of *Picea glauca* and *Picea mariana* possess, in comparison with Pycnogenol, similar concentration in phenolic compounds but are about 2 to 3 times more active considering ORAC values and about 3 times more active considering cell-based assay. *Pinus banksianna*, *Pinus resinosa* and *Larix laricina* possess similar antioxidant activity to Pycnogenol. Finally, *Abies balsamea* reveals lower antioxidant activity and total phenolic content.

## 4. Conclusions

In conclusion, ultrasonic extraction of barks of boreal forest conifers yielded extracts that are not toxic, rich in phenolic compounds and having a strong antioxidant activity. Ultrasonic assisted extraction yields and phenolic contents of bark extracts from *Picea mariana* and *Pinus banksiana* were higher than those reported in literature using reflux and maceration extraction methods. Among the seven species studied, only bark extracts of *Pinus resinosa*, *Picea glauca*, and *Picea mariana* shown phenolic contents similar or higher than Pycnogenol, a standardized commercial extract from *Pinus maritima* bark. The best overall antioxidant activities with both cellular and ORAC assays were obtained with *Picea glauca* and *Picea mariana*, and appear to vary less in regard to extraction solvent conditions used. A good correlation between total phenolic content and antioxidant activity is found for all water extracts of barks, suggesting that phenolic compounds are responsible for the activity.
